# Annotations

**Published:** 1890-10-18

**Authors:** 


### Annotations.

**Poor Men's Houses.**

All who have had anything to do with the enforcement of the sanitary laws in this country are conscious of the needless suffering and misery caused to the poor in respect to their homes. In crowded cities a class of men live by purchasing old dwelling-houses, and then farming them out to poor families at an exorbitant rental. Many of these tenement houses are quite unfit for occupation; but this class of landlord usually selects a quarter where the circumstances are such as to render it difficult, if not impossible, to close or demolish the houses though they may be unfit for human dwellings. Several of these landlords seek election on the sanitary committees in order to promote their own interests against the poor. The Artizans' Dwellings Act of last session has been enacted in the hope of diminishing or entirely removing many existing evils. It is so drawn as to enable the County Councils to come down on the rural sanitary authorities and to compel them to take the necessary proceedings for the closing and demolition of insanitary dwellings, and the removal of buildings conducive to making other buildings in a condition unfit for human habitation. When a rural sanitary authority is in default the County Council has the power to itself undertake the necessary measures at the expense of the sanitary authority. There can be no doubt that many material improvements can be effected in the present position of the dwellings of the labouring classes by a strenuous and judicious exercise by County Councils of these powers. Mr. Ritchie has done well to call the attention of the County Councils to these facts, and to express a hope that they will fully realise the responsibility which has devolved on them of carefully considering all representations, complaints, or information forwarded or submitted to them, with a view to their taking such steps as are contemplated by the Act of 1890, in cases where the rural sanitary authorities have failed to perform their duties. Bad landlords and neglectful medical officers of health should both disappear under the new legislation, if only County Councils are in earnest and set to work with a will.

**The Hospitals and the Pension Fund.**

The recent agitation concerning the treatment of nurses in hospitals may do good in many ways, despite the indefensible action of some of those concerned in it. Few will question that the wages of nurses are, on the whole, too low, and although their comfort and happiness are much more considered than they used to be, still much may yet be done to the advantage of the institutions and of the nursing staff. It is gratifying to know that the medical officers are taking an interest in the nurses, and are using their influence with the committees, to induce them to affiliate with the National Pension Fund for Nurses on the half premium plan. Many of the principal hospitals have already adopted this course, and we believe that the movement is extending at the present time; for it must be to the advantage of every hospital to secure a staff who are absolutely independent and self-respecting. The Pension Fund affords every committee an opportunity of attaching their staff to the institution by the payment of a relatively small sum well within the means of every voluntary hospital. In the few cases where the expenditure always exceeds the income we would recommend those interested in the nurses to institute a special fund with an honorary secretary or secretaries, in the same way as the special funds were formerly established to maintain a chaplain in the various hospitals. In provincial institutions the nursing staff does not usually exceed twenty or twenty-five members, so that £100 a year would be amply sufficient to enable most of these institutions to affiliate with the National Pension Fund on the half-premium principle. The plan is to grant two policies, one to the nurse in respect of her payments, and the other to the hospital in respect to its payments. All payments to the Pension Fund should be made on the returnable scale, and Table F, which includes sick pay, is not only the best value for the money, but the one best adapted to suit the circumstances of all nurses. If sick pay be entered for at the outset, then every nurse may consider herself quite independent and able to choose for herself any special work in any portion of the nursing-field which may be most congenial to her tastes and qualifications. We would counsel the medical staff of hospitals and the committees to look into this question, and to write to the Manager, 8, King Street, Cheapside, E.C., asking him for a copy of the speech of the Prince of Wales at Marlborough House, and also for a list of the institutions who have already affiliated. Those committees who lag behind and neglect to recognise the claims which the nursing staff have upon them for reasonable assistance and encouragement will find that their general funds will tend to diminish rather than to increase, because the public will come to regard them as indifferent to the best interests of those whose welfare it is their duty to promote and encourage in every way. More mischief is done in these matters by want of thought than by want of heart, and a word to the wise at this season may not fail to be useful in its effect, whilst it may prove to the advantage of all concerned.

**Transplanting a Hospital.**

There are few things more contradictory than the unconscious affection engendered by long association. A man may spend many years of his life in an ill-ventilated and unattractive office, the defects of which he recognises and resents. Circumstances may compel an individual or a family to occupy a small house or apartments inadequate to their requirements, and altogether uninviting. Others may grumble at the irksomeness of an occupation, the ties of a particular business, or the worries incidental to the daily round. Still in each case when the time arrives for a change of surroundings of circumstances or associates, poor human nature asserts itself and few people can take up the new position or occupy the convenient office without a certain sense of loss and regret. On similar grounds alone can regret be felt at the decision of the governors of the General Hospital, Birmingham, to transplant that institution from the heart of the town to a convenient and salubrious site elsewhere. Situated in Summer Lane, surrounded by large works, located in buildings so old as to be relatively insanitary to say the least, a change of site has in reality become an imperative duty. Yet those of us who are old enough to remember the General Hospital, the good work it has done, and the difficulties it has overcome, must confess to a feeling of regret that our old friend is about to take unto itself a new face. Birmingham owes much to the late Miss Ryland, but its sense of indebtedness should be enhanced tenfold by the nature of her bequest, which had annexed to it the condition that the General Hospital should be rebuilt on a new site if the £25,000 was given over to the hospital trustees. There is reason to hope that the example of Birmingham may commend itself to the anxious consideration of more than one metropolitan hospital committee. Some of the oldest and largest hospitals occupy inadequate or objectionable sites which cramp the work of the institutions, and render them hygienically much too insanitary for the abode of those who are seriously sick. We have never been able to understand how it is that some wealthy millionaire has not been moved to purchase all the buildings behind University College Hospital for example, in order to make a free gift of them and the site to the hospital authorities. If this were done, the whole site might be cleared and a hospital might be erected which could be made so sanitary as to be a model of completeness, whilst due regard is paid not only to the requirements of the patients, but to the ever-increasing claims of a medical school of the first rank. We have prepared plans of the sites of certain of the London hospitals, and should be glad to show them to any wealthy philanthropist who wishes to expend a good round sum in charity to the best advantage of his day and generation.

